# FBXO2 Promotes Proliferation of Endometrial Cancer by Ubiquitin-Mediated Degradation of FBN1 in the Regulation of the Cell Cycle and the Autophagy Pathway

**DOI:** 10.3389/fcell.2020.00843

**Published:** 2020-08-31

**Authors:** Xiaoxia Che, Fangfang Jian, Ying Wang, Jingjing Zhang, Jian Shen, Qi Cheng, Xi Wang, Nan Jia, Weiwei Feng

**Affiliations:** ^1^Department of Obstetrics and Gynecology, Ruijin Hospital, School of Medicine, Shanghai Jiao Tong University, Shanghai, China; ^2^Obstetrics and Gynecology Hospital, Fudan University, Shanghai, China; ^3^Department of Obstetrics and Gynecology, Yidu Central Hospital of Weifang, Weifang, China

**Keywords:** endometrial carcinoma, FBXO2, FBN1, ubiquitination, cell cycle, autophagy

## Abstract

F-box proteins, as substrates for S phase kinase-associated protein 1 (SKP1)-cullin 1 (CUL1)-F-box protein (SCF) ubiquitin ligase complexes, mediate the degradation of a large number of regulatory proteins involved in cancer processes. In this study, we found that F-box only protein 2 (FBXO2) was up-regulated in 21 endometrial carcinoma (EC) samples compared with five normal endometrium samples based on our Fudan cohort RNA-sequencing. The increased FBXO2 expression was associated with tumor stage, tumor grade, and histologic tumor type, and poor prognosis based on The Cancer Genome Atlas (TCGA) database. FBXO2 knockdown inhibited EC cell proliferation, and FBXO2 overexpression promoted the parental cell phenotype *in vivo* and *in vitro*. Fibrillin1 (FBN1) was also identified as a substrate for FBXO2 using a ubiquitination-proteome approach. In addition, promotion of EC proliferation by FBXO2 was regulated by specific proteins of the cell cycle (CDK4, CyclinD1, CyclinD2, and CyclinA1) and the autophagy signaling pathway (ATG4A and ATG4D) based on RNA sequencing (RNA-seq). We concluded that FBXO2 acts as an E3 ligase that targets FBN1 for ubiquitin-dependent degradation, so as to promote EC proliferation by regulating the cell cycle and the autophagy signaling pathway. Targeting FBXO2 may represent a potential therapeutic target for EC.

## Introduction

Endometrial carcinoma (EC) is the commonest gynecologic malignancy in developed countries and the fourth most common cancer in women worldwide, and its prevalence continues to increase (Morice et al., 2016). It is estimated that there will be 61,880 new cases and 12,160 deaths in the United States from EC in 2019 (Siegel et al., 2019). Therefore, it is of paramount importance that we seek new biomarkers to improve the health and survival of patients with EC.

Ubiquitination by the ubiquitin-proteasome system (UPS) is a post-translational modification that regulates various cellular processes. It is carried out by a three-step cascade of ubiquitin transfer reactions (activation, conjugation, and ligation) regulated by ubiquitin-activating enzymes (E1), ubiquitin-conjugating enzymes (E2), and ubiquitin ligases (E3), respectively (Vucic et al., 2011). F-box proteins are the substrate adaptors of S phase kinase-associated protein 1 (SKP1)-cullin 1 (CUL1)-F-box protein (SCF) ubiquitin-ligase complexes that mediate the degradation of a number of regulatory proteins involved in cancer processes including cell growth, cell division, signaling responses, and cell survival and death (Skaar et al., 2013). F-box proteins can be classified into three families based on the presence of recognizable domains beyond the F-box domain (Cenciarelli et al., 1999; Ilyin et al., 2002). Members of this family have been shown to be essential in the regulation of cell proliferation and to exhibit oncogenic or tumor-suppressive activities or in cancer drug resistance (Uddin et al., 2016; Yan et al., 2019; Liu et al., 2020). Inactivation of FBXW7 resulted in the formation of precancerous lesions and well-differentiated endometrioid adenocarcinomas (Cuevas et al., 2019). High expression of SKP2 has been reported to be correlated with poor prognosis in endometrial endometrioid adenocarcinoma (Kamata et al., 2005).

F-box only protein 2 (FBXO2) also named Fbx2, Fbs1, and NFB42 is a cytoplasmic protein and ubiquitin ligase F-box protein with specificity for high-mannose glycoproteins (Yoshida et al., 2002). FBXO2 also was reported to act as a posttranscriptional regulator of hepatic insulin signaling and might constitute a novel therapeutic target for treating metabolic disorders (Liu et al., 2017). FBXO2 was found to regulate the EMT signaling pathway in gastric cancer and colorectal cancer suggesting that its importance involved in cancers (Sun et al., 2018; Wei et al., 2018). These investigators showed that F-box proteins play an essential role in cancers, and may provide potential strategies with which to treat them. However, whether FBXO2 plays a role in EC remains poorly understood.

In this study, we report our novel findings on the important role of FBXO2 in EC progression and investigate the underlying mechanisms of its action. We hypothesize that FBXO2 can serve as a potential therapeutic target for the precise treatment of EC.

## Materials and Methods

### Clinical Specimens

This present study was approved by the ethics committee of Ruijin Hospital affiliated to Shanghai Jiao Tong University School of Medicine and Obstetrics and Gynecology Hospital of Fudan University. Twenty-one EC samples (three with clear cell carcinoma, five with serious carcinoma, and 13 with endometrioid endometrial carcinoma) and five normal samples (all of proliferative endometrium) from the Obstetrics and Gynecology Hospital, Fudan University (the “Fudan Cohort”) were used to perform RNA sequencing (RNA-seq). Tissue microarrays containing 90 paired formalin-fixed and paraffin-embedded specimens (endometrioid endometrial carcinoma and para-carcinoma tissues) from tissue representing different stages of EC in patients who underwent surgical resection at Ruijin Hospital affiliated to Shanghai Jiao Tong University School of Medicine between 2013 and 2017 were examined. Among those samples, we extracted RNA from 19 normal endometrium and 47 EC tissues for subsequent validation by qRT-PCR. We also extracted proteins from 6 normal endometrium and six EC tissues for western immunoblotting analysis.

### EC Cell Lines and Regents

AN3-CA, HEC-1B, ECC-1, KLE, and Ishikawa were kindly provided by Jie Jiang professor, Qilu Hospital of Shandong University. HEK-293T was kindly provided by Yingjie Xu professor, Ruijin Hospital of Shanghai Jiao Tong University. HEC-1A and RL95-2 cells were obtained from Chinese Academy of Science^1^. AN3-CA, HEC-1B, ECC-1, and KLE were maintained in RPMI-1640, Ishikawa and HEK-293T in DMEM, HEC-1A in McCoy’s 5A, RL95-2 in F12 medium supplemented with 10% fetal bovine serum (FBS, Gibco Inc., Albany, NY, United States), 100 μg/ml penicillin/streptomycin and 2 mM L-glutamine in a humidified incubator at 37°C and 5% CO2. All cells tested negative for mycoplasma contamination and the certificate of STR analysis were included for all cell lines. 3-Methyladenine (3-MA) (Sigma-Aldrich, St. Louis, MO, United States), palbociclib (MedChemExpress, Trenton, NJ, United States), MG132 (Selleckchem, Houston, TX, United States, S2619), and cycloheximide (CHX) (Enzo, ALX-380269) were purchased from the mentioned manufactures.

### Establishment of FBXO2 Stable Knockdown and Overexpressing Cells, and FBN1 Stable Knockdown Cells and FBN1 Transient Transfection

For the construction of stable FBXO2 knock down cell line, we first selected three interfering plasmids to test their efficiency both in mRNA and protein levels shown in Figure 2A. And we selected the shFBXO2-1 and shFBXO2-2 to establish stable cell lines. We used two shRNAs (shFBXO2-1 and shFBXO2-2) in the cells phenotype (named RL95-2-shFBXO2-1 and RL95-2-shFBXO2-2 in Figure 2), in the following mechanism and *in vivo* sections, we chose the most effective shRNA (shFBXO2-2) for further study (named RL95-2-shFBXO2 in Figures 3–7). The FBXO2, FBN1, and FBXO2/FBN1 stable knockdown cell lines (RL95-2-shFBXO2-1, RL95-2-shFBXO2-2, RL95-2-shFBN1, and RL95-2-shFBXO2/shFBN1) were established by lentiviral-based stable shRNA subcloned into the RNAi pLenti hU6-MCS-CMV-zsGreen1-PGK-Puro vector (LncBio Co., Shanghai, China) (shFBXO2-1 target sequence: TGGTGTGACGTGGAGCATGGT; shFBXO2-2 target sequence: GGAGTTCACCCACGATGAGAG; shFBXO2-3 target sequence: TCGTGGTGAAGGACTGGTACT; shFBN1 target sequence: CAGCTGGCATCAGATGGACGTTATT). Non-target control shRNA served as a negative control (RL95-2-NC). The FBXO2 stably overexpressing cell line (Ishikawa-ovFBXO2) was established by lentiviral-based stable LV-FBXO2 subcloned into the GV492 Ubi-MCS-3FLAG-CBh-gcGFP-IRES-puromycin vector (GeneChemBio Co., Shanghai, China). The transient overexpressing of FBN1 cell lines (RL95-2-ovFBN1 and Ishikawa-ovFBN1) was conducted using FBN1 plasmids cloned into a pcDNA3.1 vector (Target sequence: GAACAAAAACTCATCTCAGAAGAGGATCTG).

### Plasmid Construction, Transfection, and Immunoprecipitation

Flag-tagged wild-type (WT), truncated, and mutant (MUT) FBXO2 (NM_012168), and Myc-tagged FBN1 (NM_000138) were subcloned into the pcDNA3.1 vector (LncBio Co., Shanghai, China). HEK-293T cells were transfected with the plasmids using Lipofectamine 3000 (Invitrogen, Thermo Fisher Scientific) according to the manufacturer’s instructions for FBXO2 and FBN1 binding analysis. We incubated protein A/G agarose with antibodies (5–10 μg) for 20 min, lysed the cells, and incubated the supernatants with the protein A/G agarose and antibodies for 1 h at room temperature. The incubation was boiled with 1 × Laemmli buffer for 10 min at 99°C. The immune complexes were subjected to SDS-PAGE and analyzed by immunoblotting. To immunoprecipitate the endogenous proteins, cells were lysed with 1 × cell lysis buffer. The supernatant was precleared with protein A/G agarose with indicated antibody overnight (FBXO2, Santa Cruz Biotechnology, sc-393873; FBN1, LifeSpan BioSciences, LS-B5512). Thereafter, the incubation was boiled with 1 × Laemmli buffer and analyzed by SDS-PAGE.

### Subcutaneous Tumor Implantation Model

In total, 5 × 10^6^ (0.1 ml) RL95-2-NC, RL95-2-shFBXO2, RL95-2-shFBN1, and RL95-2-shFBXO2/shFBN1 cells were injected subcutaneously into 4-week-old immune-deficient BALB/c-nu mice (Shanghai LC Company). Tumor formation and mice weight was measured every 3 days, and tumor volume was calculated as 1/2 × length × width^2^ for almost 4 weeks. All procedures were approved by the Animal Ethic Review Committee of Shanghai Jiao Tong University School of Medicine. All animals were handled according to the Guide for the Care and Use of Laboratory Animals’ and the Principles for the Utilization and Care of Vertebrate Animals.

### Reverse Transcription-Quantitative Polymerase Chain Reaction (qRT-PCR)

Total RNA was extracted from EC cell lines and patients tissue via TRIzol (Thermo Fisher Scientific Inc., MA, United States) according to the manufacturer’s instrument. Reverse transcribe mRNA to cDNA and real-time PCR were performed by the PrimeScript^TM^ Reagent Kit and SYBR Premix Ex Taq^TM^ II (Takara Bio Inc., Shiga, Japan). The primers were synthesized from BioTNT (Shanghai BioTNT Co., Ltd.). The primers sequence of the following genes are described in Table 1.

**TABLE 1:** The primers sequence in the article.

Primer	Sequence (5′ to 3′)
FBXO2-F	ACTTGGAAGGCTGGTGTGAC
FBXO2-R	TCAAAGGAGGAGGCGAAGTA
FBN1-F	TGCCGCATATCTCCTGACCTCTG
FBN1-R	TAGCCTTCGTCACACTTGCATTCG
GAPDH-F	GGGAAGGTGAAGGTCGGAGT
GAPDH-R	GGGGTCATTGATGGCAACA
CCND1-F	GAGACCATCCCCCTGACGGC
CCND1-R	TCTTCCTCCTCCTCGGCGGC
CCND2-F	CATCCTCACGGCCTCGGCT
CCND2-R	CGGCGTGTGTTTATCGGAATCCA
THBS1-F	AGACTCCGCATCGCAAAGG
THBS1-R	TCACCACGTTGTTGTCAAGGG
CHEK1-F	TCTTTGGACTCGCTCAAGAAGCCT
CHEK1-R	ATTTCAACCTTCGGTGTGCTTGGG
CDK4-F	GAAGAAG AAGCGGAGGAAGAGG
CDK4-R	TTAGGTTAGTGCGGGAATGAAT
SMC1A-F	AAAGCATCAAGCGCCTTTAC
SMC1A-R	GCTGGCATAGGTCAATGAGG
MCM7-F	CCTACCAGCCGATCCAGTCT
MCM7-R	CCTCCTGAGCGGTTGGTTT
CDC14B-F	CAAACGCTTTACGGATGCTGG
CDC14B-R	TGATGTAGCAGGCTATCAGAGT
PPP2CA-F	GATCTTCTGTCTACATGGTGGTCTC
PPP2CA-R	ACACATTGGACCCTCATGGGGAA
ATG4A-F	TTGGCCCAGGATGACAGCTG
ATG4A-R	AGGGCCCGTTCCACCAATTG
ATG4B-F	TGAGTCTTGTGGTGTGTGGT
ATG4B-R	TACTTTCCCAGGACAGGCAG
ATG4C-F	GTTACCTGCAGAGTCGGGAT
ATG4C-R	GGCCAGTTCTCAATGTGCAG
ATG4D-F	GTCCATGAACTCAGTGTCGC
ATG4D-R	GAACTTGTCCACTTCGTCCG

### Immunofluorescence (IF) Staining

Ishikawa and RL95-2 cells were incubated with anti-FBXO2 (1:50, Santa Cruz Biotechnology, sc-398111) and anti-FBN1 antibodies (1:100, Thermo Fisher Scientific, PA5-82743) in PBS at 4°C overnight, and then with Alexa Fluor 488-conjugated and 555-conjugated donkey anti-goat secondary antibody (1:1000, Abcam; 1:1000, Life Technology). Nuclei were stained with 4,6-diamidino-2-phenylindole (DAPI) (Sigma, D9542) for 10 min. Confocal microscopy (Leica, Germany) was used to capture images.

### Tissue Microarray Assay Staining

Tissue microarrays containing 90 paired formalin-fixed and paraffin-embedded specimens of EC were obtained from Ruijin Hospital affiliated to Shanghai Jiao Tong University School of Medicine. Slides of patients tissue were incubated with anti-FBXO2 rabbit polyclonal (1:100, Abcam, ab230307), anti-FBN1 rabbit polyclonal (1:50, Arigobio, ARG66654), and Ki67 (1:100, GB13030-2, Goodbiotechnology, Wuhan, China) antibodies. We assessed the expression levels of FBXO2 and FBN1 by the percentage of positively stained cells, and the IHC index was determined on the basis of the staining intensity and the percentage of immuno-reactive cells. Staining intensity was rated as 0 (negative), 1 (weakly positive), 2 (moderately positive), or 3 (strongly positive), and the percentage of staining was rated as 0 (0%), 1 (1-25%), 2 (26-50%), 3 (51-75%), or 4 (76-100%). The IHS (immunohistochemistry score) was calculated by multiplying the quantity and intensity scores ranging from 0 to 12. An IHS score of 0–6 was low and 7–12 was high immunoreactivity as previously described (Zhi et al., 2010). The expression of FBXO2, FBN1, and Ki67 in mouse xenografts was quantified using Image Pro Plus (IPP), involving the three parameters of integrated optical density (IOD), area, and mean optical density were involved: IOD sum/area. Images were obtained with a microscope (Olympus BX53; Olympus, Tokyo, Japan) fitted with a digital camera (Olympus DP73; Olympus). Five randomly selected images at ×400 magnification of each sample were taken to achieve a mean optional density value with Image Pro-Plus 6.0 (version 6.0.0.206; Media Cybernetics, Inc., Bethesda, MD, United States).

### Flow Cytometry for Analysis of Cell Cycle and Apoptotic Cells

To analyze the cell cycle, cells were seeded in six-well culture plates and cultured to 90% confluence and were harvested and permeabilized with cold 75% ethanol at −20°C overnight. Then the cells were centrifuged at 150 × *g* for 10 min at room temperature, the precipitate was resuspended in 2 ml of 0.9% physiologic saline and centrifuged at 150 × *g* for 10 min. Cell cycle progression was evaluated after propidium iodide (PI) staining for 30 min in the dark.

We identified apoptotic cells using the Annexin V/Dead Cell Apoptosis Kit (BD-Pharmingen). Briefly, cells were seeded in 6-well plates for 48 h to 95% confluence. Then cells were harvested and washed twice with ice-cold PBS resuspended in 1× annexin-binding buffer and incubated with 5 μl of Annexin V-PE (phycoerythrin) and 5 μl of 7-AAD (7-amino-actinomycin) at room temperature for 15 min in the dark. PE and 7-AAD fluorescence was analyzed by flow cytometry within 1 h.

### Proliferation Potential Analysis

#### Colony Formation Assay

Ishikawa-NC, Ishikawa-ovFBXO2, RL95-2-NC, RL95-2-shFBXO2, RL95-2-shFBN1, and RL95-2-shFBXO2/shFBN1 cells were seeded in six-well plates at 600, 5000 cells/well, respectively, and then incubated for 12 days as previously in Che et al. (2016).

#### Cell Counting Kit-8 (CCK-8) Assay

Cell proliferation was also measured by the CCK-8 assay kit (Dojindo Japan). Briefly, cells with 4000–10,000 density were seeded in 96-well culture plates for 6–7 days. After incubation for indicated time, 10 μl CCK-8 reagent was added to each well for incubating 1 h and the absorbance was measured at 450 nm.

#### A EdU Assay

A EdU assay (Riobio, Guangzhou, China) was used in proliferation assays performed in a 96-well format with 4,000 and 10,000 cells of Ishikawa and RL95-2 cell lines, respectively, according to the manufacturer’s instrument. Images were captured by fluorescence microscope.

#### A Real-Time Cell Proliferation Assay (RTCA)

Proliferation assays were also performed in 16-well CIM plates in an xCELLigence DP instrument for real-time cell proliferation analysis (Roche, Mannheim, Germany). Cells (Ishikawa: 4,000 cells/well, and RL95-2: 10,000 cells/well) were seeded in the plate and grow on top of electrodes so that the impedance varies on the basis of the number of cells attached and the quality of cell-electrode interaction. Electrode impedance, displayed as cell index, was used to monitor cell viability.

### RNA Sequencing Analysis

The RNA expression profile was determined in the sequencing libraries generated form a NEBNext^®^ Ultra^TM^ RNA Library Prep Kit for Illumina^®^ (NEB, United States). The clustering of the samples were performed on a cBot Cluster Generation System using TruSeq PE Cluster Kit v3-cBot-HS (Illumina), and the library preparations were sequenced on an Illumina NovaSeq platform. Gene ontology (GO), Kyoto Encyclopedia of Genes and Genomes (KEGG) pathway enrichment analysis, and Gene Set Enrichment Analysis (GSEA) were performed to analyze the FBXO2 regulated genes based on the sequencing data sets. The RNA-seq data was available in Supplementary Data named “21 EC VS 5 normal samples diff genes” and “shFBXO2 VS Negtive Control diff genes.”

### K-ε-GG Profiling and Proteome Analysis by Liquid Chromatograph-Tandem Mass Spectrometry (LC MS/MS)

The supernatant from each sample, containing precisely 10 mg of protein was digested with Trypsin Gold (Promega) at a 1:50 enzyme-to-substrate ratio. After 16 h of digestion at 37°C, peptides were desalted with a C18 cartridge to remove the high urea, and desalted peptides were dried by vacuum centrifugation. MOPS IAP buffer (50 mM MOPS, 10 mM KH_2_PO_4_, and 50 mM NaCl) was added to resuspend the lyophilized peptides. Then centrifuged peptides for 5 min at 12000 *g*. Supernatants were mixed with anti-Ubiquitin Remnant Motif K-ε-GG beads (CST #5562, Cell Signaling Technology) for 2.5 h at 4°C. Beads were washed in MOPS IAP buffer, and then in water, prior to elution of the peptides with 0.15% TFA. In preparation for analysis, the peptides were desalted using peptide desalting spin columns (Thermo Fisher, 89852).

We performed shotgun proteomics analyses using an EASY-nLC^TM^ 1200 UHPLC system (Thermo Fisher) coupled with an Orbitrap Q Exactive HF-X mass spectrometer (Thermo Fisher) operating in the data-dependent acquisition (DDA) mode, and the raw data were processed using [the Proteome Discoverer 2.2 (PD) 2.2, Thermo] search engines. Tandem mass spectra were searched against the Swissprot human (20,274 sequences) database concatenated with a reverse decoy database. The K-ε-GG profiling and proteome analysis by liquid chromatograph-tandem mass spectrometry was performed in Novogene Co., Ltd., Beijing, China. The proteome sequencing data was available in Supplementary Data named “ishikawa ov VS Ishikawa nc diff proteins.”

### Statistical Analysis

Statistical analysis was performed using IBM SPSS 22.0 software (SPSS Inc., Chicago, IL, United States). The data all are presented as mean ± standard deviation (SD). Student’s *t*-test and one-way analysis of variance were applied to assess the differences. The enumeration data was evaluated using χ^2^ test or Fisher exact-probability test. The cumulative survival curves were drawn using the Kaplan-Meier method. *P* < 0.05 was considered to be statistically significant.

## Results

### FBXO2 Is Up-Regulated in Endometrial Cancer and Is Correlated With Tumor Progression

**FIGURE 1:**
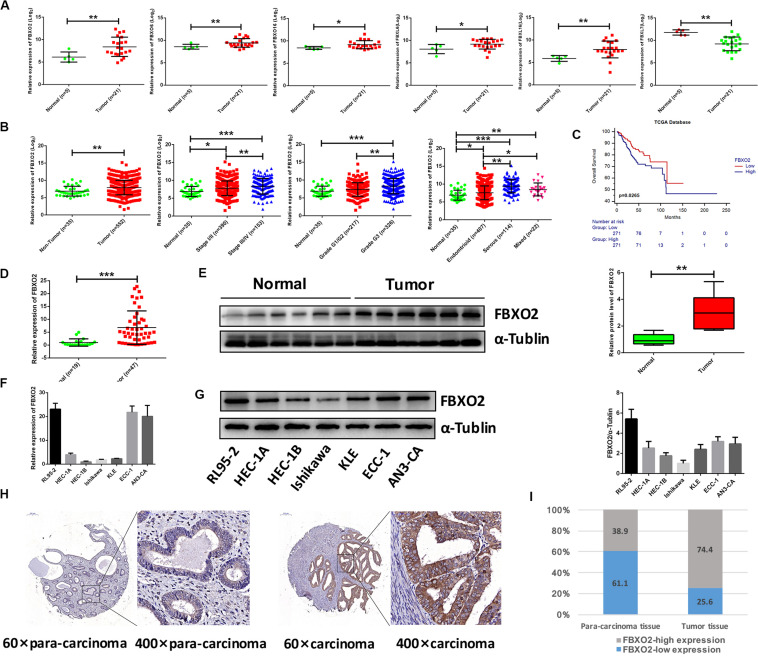
Frequent dysregulation of the F-box family in human EC based on the RNA-sequencing data in the Fudan cohort, and the frequent up-regulation of FBXO2 in the TCGA database, and in human EC tissues. **(A)** The mRNA expression of F-box family members (FBXO2, FBXO6, FBXO16, FBXL6, FBXL16, and FBXL7) in our RNA-sequencing data (*P < 0.05, **P < 0.01, *t*-test). **(B)** FBXO2 mRNA expression in 552 EC samples compared with 35 non-tumorous samples, based on the TCGA database (**P < 0.01, *t*-test); FBXO2 mRNA expression at different stages, grades and histologic types of ECs (*P < 0.05, **P < 0.01, and ***P < 0.001, one-way ANOVA). **(C)** The Kaplan-Meier survival analysis of EC patients with different expression levels of FBXO2 (*P* = 0.0265, log-rank test). **(D)** FBXO2 mRNA expression in 47 EC samples compared with 19 normal samples (***P < 0.001, *t*-test). **(E)** Western immunoblotting analysis of FBXO2 protein expression in six EC samples compared with six normal samples (**P < 0.01, *t*-test). **(F,G)** FBXO2 mRNA **(F)** and protein **(G)** expression in seven EC cell lines. **(H)** Immunohistochemical staining of FBXO2 in 90 EC samples and paired para-carcinoma samples. Representative photomicrographs from a patient. Original magnification, ×60 and ×400; scale bars: 200 and 50 μm, respectively. **(I)** Proportions of low or high expression in EC samples and para-carcinoma samples.

To explore the mechanisms underlying the progression of EC, we performed RNA-seq analysis on normal endometrium (*n* = 5) and EC samples (*n* = 21). A total of 918 genes were differentially expressed (641 up- and 277 down-regulated genes) in EC tissues compared with normal endometrium (with a padj < 0.01 and | Log2Fold change| > 2). F-box protein family genes were significantly enriched in our sequencing results, including FBXO2, FBXO6, FBXO16, FBXL6, FBXL16, and FBXL7 (Figure 1A), and we chose FBXO2 for subsequent experiments as it was the most significantly up-regulated gene.

Consistent with our data, FBXO2 mRNA was increased in the TCGA database that consisted of 552 patients with endometrial cancer (Figure 1B). When we then divided the EC samples into different components according to the EC stage, grade, and histologic type. The results showed that FBXO2 expression in stage III/IV samples and grade 3 was higher than that in stage I/II and grade 1/2, respectively (Figure 1B). We also found that the FBXO2 expression in the serous type was significantly higher than in the endometrioid type (Figure 1B), which implied that FBXO2 high expression was related to EC progression. Moreover, high level of FBXO2 expression was significantly associated with shorter survival (Figure 1C). FBXO2 was also up-regulated in EC samples compared with normal samples both in the mRNA and protein levels in Fudan cohort (Figures 1D,E). Furthermore, when we evaluated the expression of FBXO2 using tissue microarrays. We observed that the proportion of highly expressed FBXO2 in EC samples was significantly higher than that in para-carcinoma samples (Figures 1H,I, 74.4 vs. 38.9%, *P* < 0.05). Next, we investigated whether high FBXO2 expression was related to clinicopathologic features and found that high FBXO2 expression was significantly associated with muscular infiltration and Ki67 staining (Table 2). Our results suggested that high expression of FBXO2 in EC samples was correlated with endometrial cancer progression.

**TABLE 2:** Clinicopathological characteristics of Test trail patients according to FBXO2 expression.

Characteristics	Patients			FBXO2 expression		
	No.	%	High	Low	χ^2^	*P*-value ^*a*^
All patients	90	100	67	23		
**Age (years)**					**1**.76	0.185
<60	51	56.7	36	16		
≥60	39	43.3	31	7		
**Tumor size (cm)**					0.387	0.534
<4	64	71.1	51	13		
≥4	26	28.9	16	10		
**Lymphovascular invasion**					0.658	0.417
Positive	8	8.89	5	3		
Negative	82	91.1	62	20		
**Lymph node metastasis**					2.574	0.109
Metastasis	32	35.6	27	5		
No metastasis	58	64.4	40	18		
**FIGO stage**					1.045	0.307
I/II	73	81.1	56	17		
III/IV	17	18.9	11	6		
**Muscular infiltration**					5.711	**0.017**
<1/2	70	77.8	48	22		
≥1/2	20	22.2	19	1		
**Ki67 staining**					7.809	**0.005**
<50%	52	57.8	33	19		
≥50%	38	42.2	34	4		

*^*a*^*P*-value < 0.05 marked in bold font shows statistical significant.*

### FBXO2 Promotes Proliferation of EC Cells

**FIGURE 2:**
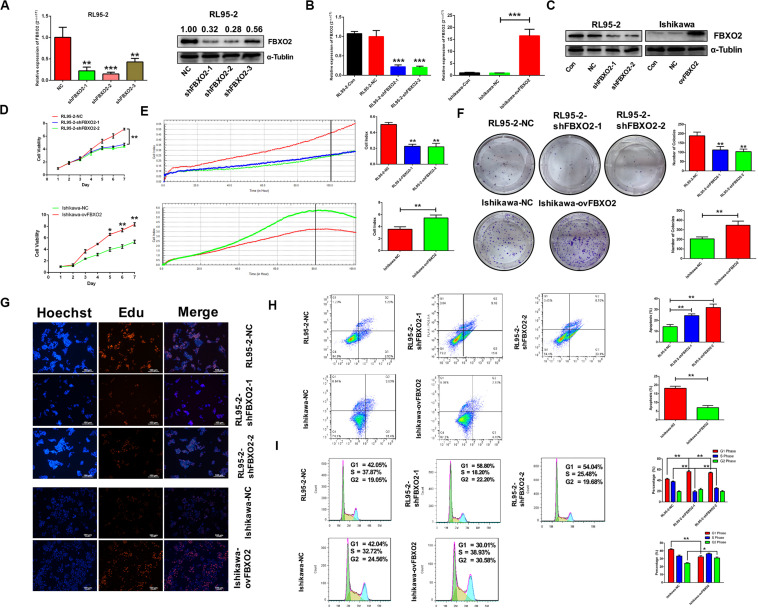
FBXO2 promotes EC cells proliferation. **(A)** Three interfering plasmids were used to test FBXO2 interfering efficiency both in mRNA and protein levels in RL95-2 cell line. Data are presented as mean ± SD, **P < 0.01, ***P < 0.001, *t*-test. **(B,C)** FBXO2 mRNA **(B)** and protein **(C)** levels in RL95-2-shFBXO2-1/2 and Ishikawa-ovFBXO2 cell lines compared with their parental cells. Data are presented as mean ± SD, ***P < 0.001, *t*-test. **(D–F)** EC cell proliferation was measured by CCK-8 assay **(D)**, RTCA **(E)**, and colony formation assay **(F)** in RL95-2-shFBXO2-1/2 and Ishikawa-ovFBXO2 cell lines compared with their parental cells. Data are presented as mean ± SD, *P < 0.05, **P < 0.01, *t*-test. **(G)** EdU staining in RL95-2-NC, RL95-2-shFBXO2-1/2, Ishikawa-NC, and Ishikawa-ovFBXO2 groups. **(H)** Apoptotic index was measured by flow cytometric assay in RL95-2-shFBXO2-1/2 and Ishikawa-ovFBXO2 cells compared with their parental cells. Data are presented as mean ± SD, **P < 0.01, *t*-test. **(I)** Cell cycle analysis by flow cytometric assay in RL95-2-shFBXO2-1/2 and Ishikawa-ovFBXO2 cells compared with their parental cells. Data are presented as mean ± SD, *P < 0.05 and **P < 0.01, *t*-test.

To explore whether FBXO2 plays a crucial role in EC progression, we selected 2 EC cell lines (RL95-2 with FBXO2 high expression, and Ishikawa with FBXO2 low expression) (Figures 1F,G) to further validate the function of FBXO2. CCK-8, RTCA, colony formation assay, and EdU staining showed that FBXO2 knockdown significantly inhibited proliferation of RL95-2-shFBXO2-1/2 cells, while overexpression in Ishikawa-ovFBXO2 cells significantly promoted proliferation (Figures 2D–G). In addition, cell cycle analysis showed that FBXO2 knockdown in RL95-2-shFBXO2-1/2 cells increased the proportion of cells in G1 phase and decreased the proportion of cells in S phase; while FBXO2 overexpression in Ishikawa-ovFBXO2 cells decreased the proportion of cells in G1 phase and increased the proportion of cells in G2 phase (Figures 2H,I). Collectively, these results suggested that FBXO2 critically regulated EC cells proliferation.

### FBXO2 Interacts With FBN1 and Negatively Regulates the Stability of the FBN1

**FIGURE 3:**
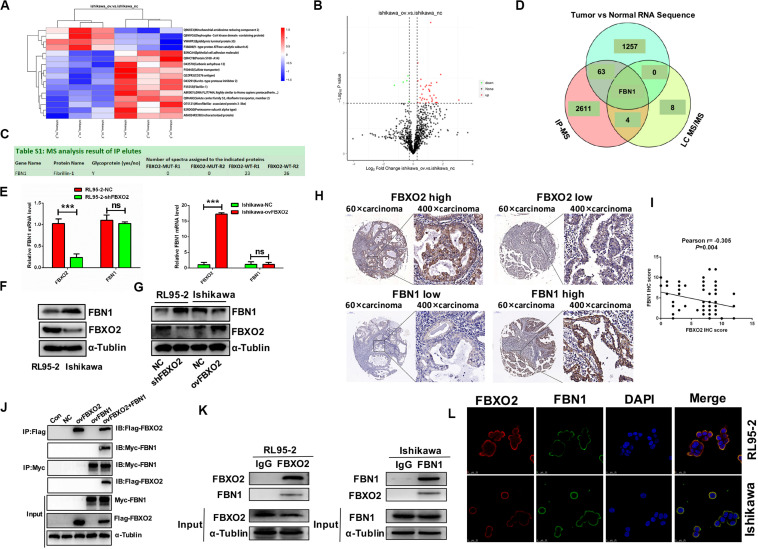
FBN1 identified as a novel substrate for FBXO2. **(A,B)** Cluster analysis and volcano plot of the differential proteins regulated by FBXO2 ubiquitination based on the LC MS/MS data. **(C)** FBN1 protein was directly bound to FBXO2 protein. **(D)** The intersection of IP-MS, LC MS/MS, and our RNA sequencing data. **(E)** FBN1 mRNA expression measured by RT-PCR in RL95-2-shFBXO2 and Ishikawa-ovFBXO2 cells compared with their parental cells (*P* > 0.05). ***P < 0.001, *t*-test. **(F)** Endogenous FBXO2 and FBN1 protein levels in RL95-2 and Ishikawa cells. **(G)** FBN1 protein levels as measured by western blot analysis in shFBXO2-RL95-2 cells and Ishikawa-ovFBXO2 cells compared with their parental cells. **(H)** Immunohistochemical staining of FBXO2 and FBN1 in 90 EC samples and paired para-carcinoma samples. Left four photographs: FBXO2 was highly expressed in carcinoma tissues (upper panel), while FBN1 showed low expression in the same patient (lower panel). Right four photographs: FBXO2 showed low expression in carcinoma tissues (upper) while FBN1 was highly expressed in the same patient (lower). Original magnification, ×60 and ×400; scale bar: 200 and 50 μm, respectively. **(I)** Inverse correlation between FBXO2 and FBN1 staining, Pearson *r* = –0.305, *P* = 0.004. **(J)** Exogenous FBXO2 and FBN1 proteins interacted with each other in HEK-293T cells. HEK-293T cells were transfected with Flag-FBXO2, Myc-FBN1, and co-transfected with Flag-FBXO2 and Myc-FBN1 for 48 h, respectively. After treatment with 20 μM MG132 for 8 h, cell lysates were prepared for co-IP with anti-Flag or anti-Myc beads and western blot analysis. **(K)** Endogenous FBXO2 and FBN1 proteins interacted with each other in endometrial cancer cell lines. RL95-2 and Ishikawa cell lysates were prepared for co-IP with anti-FBXO2 or anti-FBN1 and western blot analysis. **(L)** FBXO2 and FBN1 co-localized in RL95-2 and Ishikawa cells cytoplasm and membrane. EC cells were immunostained with anti-FBXO2 (red) and anti-FBN1 (green) antibodies and visualized with confocal microscopy. DAPI (blue) was used to indicate cell nuclei. Scale bar, 25 μM.

To identify the proteome-wide changes in ubiquitylated proteins associated with the oncogenic tumor function of FBXO2, we isolated the FBXO2 protein complex from Ishikawa-ovFBXO2 and Ishikawa-NC stable cell lines mixed with anti-ubiquitin remnant motif, and determined the proteins present in the complex by using liquid chromatograph-tandem mass spectrometry (LC MS/MS) to interrogate the differences in the proteome and ubiquitylome between Ishikawa-NC and Ishikawa-ovFBXO2 cells. As verification of the efficiency of this approach, we obtained the peptides that were ubiquitin-mediated by FBXO2 as shown in Figures 3A,B. To obtain the FBXO2-interacting proteins, Liu et al. (2017) isolated the FBXO2 protein complex from HEK293T cells expressing Flag-tagged wild-type (WT) FBXO2 or F-box-associated domain mutant (MUT) that cannot recognize glycoproteins (Mizushima et al., 2007; Glenn et al., 2008), and determined the proteins by using high-performance liquid chromatography/tandem mass spectrometry analysis (IP-MS). The intersection of IP-MS, LC MS/MS, and Fudan cohort RNA-seq results revealed the fibrillin1 (FBN1) protein. Given that the interaction between FBXO2 and FBN1 has not been previously reported, we examined the potential functional relationship between them.

To clarify the interaction between FBXO2 and FBN1, we first measured endogenous FBN1 levels and the results showed that FBN1 mRNA levels were similar in RL95-2 and Ishikawa cells (Figure 3E), however, FBN1 protein levels were higher in Ishikawa cells and lower in RL95-2 cells which was inversely correlated with endogenous FBXO2 levels (Figure 3F). In addition, after knockdown of FBXO2, FBN1 protein levels were dramatically increased in RL95-2-shFBXO2 cells, while FBN1 protein was decreased after overexpression of FBXO2 in Ishikawa-ovFBXO2 cells (Figure 3G). Tissue array (IHC) results also showed that high expression of FBXO2 was correlated with low expression of FBN1 (Figures 3H,I, Pearson *r* = −0.305, *P* = 0.004). Furthermore, exogenous and endogenous Co-IP assays were performed to confirm the direct interaction between FBXO2 and FBN1 (Figures 3J,K). FBXO2 and FBN1 also co-localized in RL95-2 and Ishikawa cell cytoplasm and membrane as observed with confocal microscopy (Figure 3L).

**FIGURE 4:**
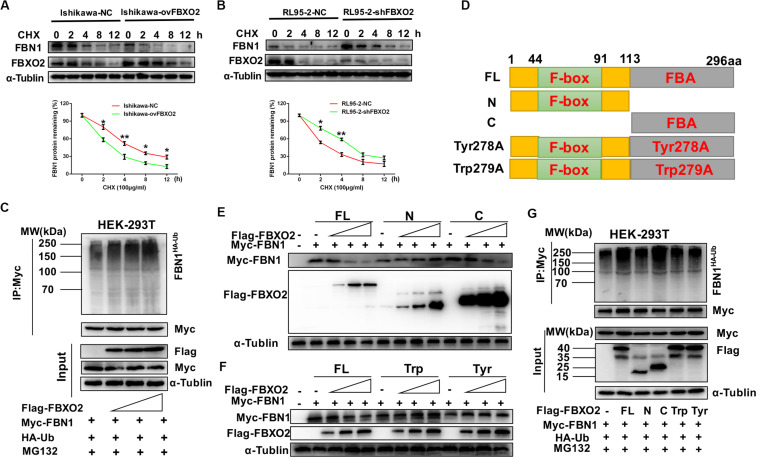
FBXO2 degrades FBN1 protein through its ubiquitination. **(A,B)** Overexpression or knockdown of FBXO2 shortened or prolonged FBN1 protein half-life in Ishikawa or RL95-2 cells, respectively. Ishikawa-NC, Ishikawa-ovFBXO2, RL95-2-NC, and RL95-2-shFBXO2 cells were treated with 100 μg/ml of the protein synthesis inhibitor cycloheximide (CHX), and harvested at indicated time points for evaluation of FBN1 protein levels. *P < 0.05, **P < 0.01. **(C)** FBXO2 enhanced FBN1 polyubiquitination in a dose-dependent manner. HEK-293T cells were transfected with Flag-FBXO2, Myc-FBN1, and HA-ub for 48 h and then treated with 20 μM MG132 for 8 h. Cell lysates were prepared for co-IP with anti-Myc beads and western blotting analysis was used to detect the HA-ub (FBN1 ubiquitination levels). **(D)** Schematic representation of the FBXO2 protein. FL, full long; N, N terminus, including F-box domain; N, N terminus, including FBA domain; Tyr278A, FBA domain was replaced with non-functional Tyr278A mutant; Trp279A, FBA domain was replaced with non-functional Trp279A mutant. **(E)** The FBXO2 C terminus down-regulated FBN1 protein levels in a dose-dependent manner while the FBXO2 N terminus did not have the function. HEK-293T cells were transfected with 1.5 μg of Myc-FBN1 and Flag-FBXO2 FL/N/C terminus plasmids at doses of 0, 0.75, 1.5, or 3.0 μg for 48 h before harvesting for western blotting analysis. α-Tublin was used as a loading control. **(F)** The FBXO2 Tyr278A and Trp279A mutants did not down-regulate the FBN1 protein levels. HEK-293T cells were transfected with 1.5 μg Myc-FBN1 and Flag-FBXO2 FL/Tyr278A/Trp279A plasmids at doses of 0, 0.75, 1.5, or 3.0 μg for 48 h before harvesting for western blotting analysis. α-Tublin was used as a loading control. **(G)** The FBXO2 C terminus (FBA domain) enhanced FBN1 polyubiquitination. HEK-293T cells were transfected with Flag-FL/N/C/Tyr278A/Trp279A, Myc-FBN1, and HA-ub for 48 h and then treated with 20 μM MG132 for 8 h. Cell lysates were prepared for co-IP with anti-Myc beads and western blotting analysis was used to detect the HA-ub (FBN1 ubiquitination levels).

Because FBXO2 interacted with FBN1, we tested whether FBXO2 regulated FBN1 stability or accelerated its protein degradation. The ubiquitination of FBN1 was also increased by ectopic expression of FBXO2 in HEK293T cells treated with MG132, a proteasome inhibitor (Figure 4C). Furthermore, overexpression of FBXO2 in Ishikawa-ovFBXO2 cells reduced the half-life of FBN1 compared with Ishikawa-NC cells while knockdown of FBXO2 in RL95-2-shFBXO2 cells augmented the half-life of FBN1 compared with RL95-2-NC cells (Figures 4A,B). These results supported the hypothesis that FBXO2 interacted with FBN1, and regulated its stability and promoted its degradation.

As FBXO2 was proven to interact with FBN1 and promote its degradation by polyubiquitination, we next conducted experiments to determine which domain of FBXO2 serves this function. The F-box associated (FBA) domain of FBXO2 is essential for its glycoprotein-recognizing activity, and this activity is completely abolished by two residues mutations named Trp279A and Tyr278A (Mizushima et al., 2007; Glenn et al., 2008). Therefore, we constructed FBXO2 FL, FBXO2 N-terminal F-box domain, FBXO2 C-terminal FBA domain and their corresponding mutant structures (FBXO2-Tyr278A, FBXO2-Trp279A) were established (Figure 4D). The results showed that the FBXO2 C terminus down-regulated FBN1 protein levels in a dose-dependent manner while the FBXO2 N terminus did not exert this function (Figure 4E). We also constructed the FBXO2 Tyr278A and Trp279A mutant plasmids and results revealed that these two mutant plasmids did not down-regulate FBN1 protein levels (Figure 4F). To prove whether the FBXO2 FBA domain regulated FBN1 polyubiquitination levels, HA-ub and Myc-FBN1 were co-transfected into HEK-293T cells with various domains of FBXO2 and enzymatic dead mutant (Tyr278A and Trp279A). Our results revealed that the FL, and normal C-terminal domain of FBXO2 enhanced FBN1 polyubiquitination (Figure 4G). These results support the concept that FBXO2, along with FBA domain activity, promoted FBN1 protein degradation through polyubiquitination of FBN1 protein.

### FBN1 Is Required for FBXO2 to Exert Functional Impacts on EC Proliferation

**FIGURE 5:**
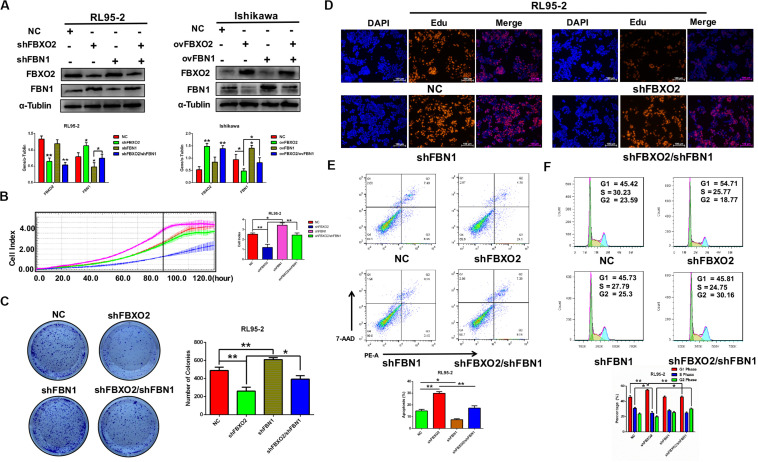
FBXO2 regulates EC proliferation through FBN1. **(A)** FBXO2 and FBN1 protein levels in RL95-2-shFBN1, and RL95-2-shFBXO2/shFBN1 stably transfected cell lines (left) and in Ishikawa-ovFBN1 and Ishikawa-ovFBXO2/ovFBN1 transiently transfected cell lines (right). *P < 0.05, **P < 0.01, one-way ANOVA. **(B,C)** Cell proliferation was measured by RTCA **(B)**, and colony formation assay **(C)** in RL95-2-shNC, RL95-2-shFBN1, and RL95-2-shFBXO2/shFBN1 stable cell lines. Data are presented as mean ± SD, *P < 0.05, **P < 0.01, one-way ANOVA. **(D)** EdU staining intensity and proportion in RL95-2-NC, RL95-2-shFBXO2, RL95-2-shFBN1, RL95-2-shFBXO2/shFBN1 stable cell lines. **(E,F)** Apoptotic index **(E)** and cell cycle analysis **(F)** as measured by flow cytometric assay in RL95-2-NC, RL95-2-shFBXO2, RL95-2-shFBN1, and RL95-2-shFBXO2/shFBN1 cell lines. Data are presented as mean ± SD, *P < 0.05, **P < 0.01, one-way ANOVA.

To investigate whether FBN1 was required for FBXO2 to regulate the proliferation of EC cells, we generated the stable FBN1 knockdown cell line RL95-2-shFBN1 and stable double FBXO2/FBN1 double-knockdown cell line RL95-2-shFBXO2/shFBN1 (Figure 5A). We found that silencing of FBXO2 in RL95-2 cells significantly inhibited cell proliferation compared with RL95-2-NC. However, silencing of both FBXO2 and FBN1 in RL95-2 cells reversed this phenotype (Figures 5B–D). Flow cytometric assay revealed that knockdown of FBXO2 caused an increased apoptotic proportion of cells compared with the RL95-2-NC group and knockdown of both FBXO2 and FBN1 decreased the apoptotic proportion compared with the RL95-2-shFBXO2 group (Figure 5E). Flow cytometric assay also showed that knockdown of FBXO2 caused an increase in the percentage of cells in the G1 phase and a decrease in cells in the S phase compared with the RL95-2-NC group. Knockdown of both FBXO2 and FBN1 revealed a decrease in the percentage of cells in the G1 phase and a concomitant increase in cells in the G2 phase compared with the RL95-2-shFBXO2 group (Figure 5F). All these results demonstrated that FBN1 was required for FBXO2 to exert functional impacts on the proliferation of EC.

### FBXO2 Regulates the Cell Cycle and the Autophagy Signaling Pathway

**FIGURE 6:**
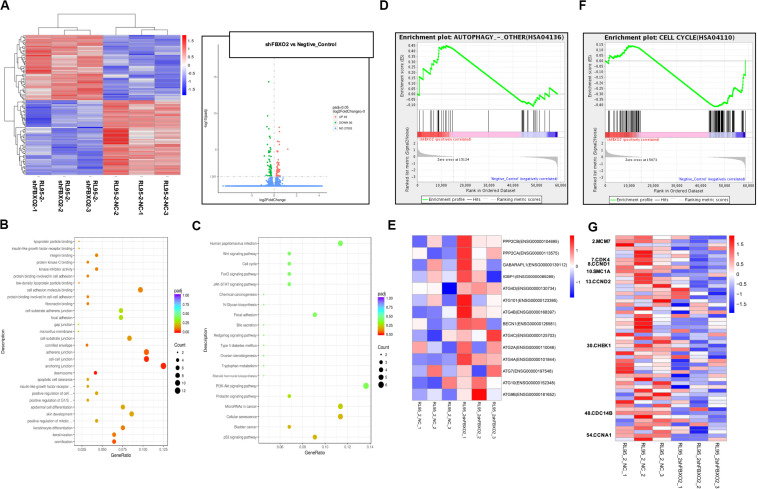
FBXO2-FBN1 promote EC cells proliferation by regulating the cell cycle and autophagy signaling pathways. **(A)** Heatmap and volcano plot of differential genes enrichment based on the RNA sequencing of the RL95-2-NC and RL95-2-shFBXO2 groups. **(B)** Go functional enrichment analysis. **(C)** KEGG pathway enrichment analysis. **(D,E)** GSEA (autophagy signaling pathway) enrichment analysis and the corresponding heatmap. **(F,G)** GSEA (the cell cycle) enrichment analysis and the corresponding heatmap.

To explore the mechanisms underlying the regulatory actions of FBXO2 on EC cells, we performed RNA-seq analysis on RL95-2-NC and RL95-2-shFBXO2 cells and identified a total of 105 genes differentially expressed in RL95-2-shFBXO2 groups (with a padj < 0.05 and |Log2Fold change| > 0) (Figure 6A). By GO and KEGG pathway enrichment analysis, these genes were principally categorized into regulation of G1/S, cell cycle, focal adhesion, PI3k-Akt pathway, Wnt pathway, and p53 pathway (Figures 6B,C). By GSEA we categorized these genes into autophagy, and cell cycle among others (Figures 6D–G).

**FIGURE 7:**
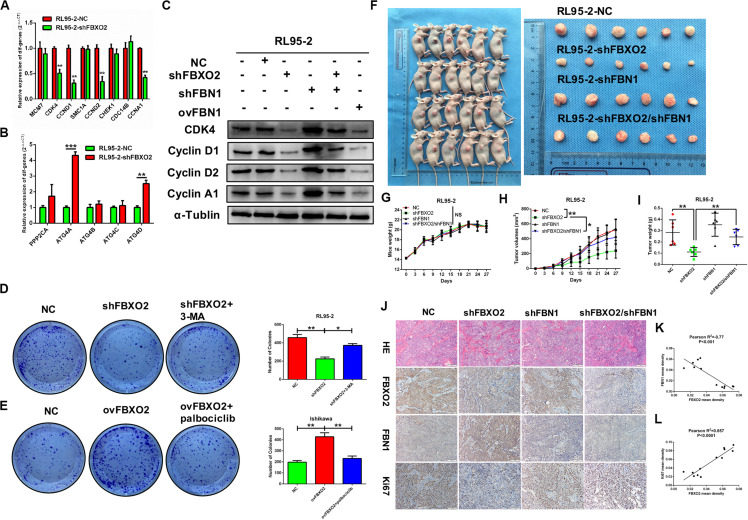
**(A–L)** Verification of differential genes associated with the cell cycle and autophagy signaling pathways. **(A)** mRNA levels of MCM7, CDK4, CCND1, SMAC1A, CCND2, CHEK1, CDC14B, and CCNA1 in RL95-2-NC and RL95-2-shFBXO2 groups. CDK4, CCND1, CCND2, and CCNA1 were significantly down-regulated in the RL95-2-shFBXO2 group compared with RL95-2-NC group. Data are presented as mean ± SD, **P < 0.01, *t*-test. **(B)** mRNA levels of PPP2CA, ATG4A, ATG4B, ATG4C, and ATG4D in the RL95-2-NC and RL95-2-shFBXO2 groups. ATG4A and ATG4D were significantly up-regulated in the RL95-2-shFBXO2 group compared with the RL95-2-NC group. Data are presented as mean ± SD, **P < 0.01, ***P < 0.001, *t*-test. **(C)** Protein levels of CDK4, CyclinD1, CyclinD2, and CyclinA1 in RL95-2-NC, RL95-2-shFBXO2, RL95-2-shFBN1, RL95-2-shFBXO2/shFBN1, and RL95-2-ovFBN1 cells. Knockdown of FBXO2 and overexpression of FBN1 revealed the same tendency in decreasing protein levels of CDK4, CyclinD1, CyclinD2, and CyclinA1 compared with the RL95-2-NC group. Knockdown of both FBXO2 and FBN1 showed increased protein levels of CDK4, CyclinD1, CyclinD2, and CyclinA1 compared with the RL95-2-shFBXO2 group. α-Tublin was used as a loading control. **(D)** The autophagy-specific inhibitor 3-MA (2 mM) reversed the effects of FBXO2 on the RL95-2 cells proliferation. Data are presented as mean ± SD, *P < 0.05, **P < 0.01, one-way ANOVA. **(E)** The CDK4/6 inhibitor palbociclib (2 μM) reversed the effects of FBXO2 on the Ishikawa cells proliferation. Data are presented as mean ± SD, **P < 0.01, one-way ANOVA. **(F–L)**, FBXO2 silencing inhibited EC carcinogenicity *in vivo* via FBN1. **(F)** RL95-2-NC, RL95-2-shFBXO2, RL95-2-shFBN1, and RL95-2-shFBXO2/shFBN1 cells were injected into BALB/c nude mice subcutaneously (0.2 ml, 5 × 10^6^ cells) and harvested at day 27. **(G)** Mice weight were monitored every 3 days and recorded (*P* > 0.05). **(H,I)** Tumor weight was recorded in a time-dependent manner **(H)** and at harvest day **(I)** among the RL95-2-NC, RL95-2-shFBXO2, RL95-2-shFBN1, and RL95-2-shFBXO2/shFBN1 groups. Data are presented as mean ± SD, *P < 0.05, **P < 0.01, one-way ANOVA. **(J)** IHC staining for FBXO2, FBN1, and Ki67 in histologic sections of transplanted tumors. **(K)** Inverse correlation between FBXO2 and FBN1 staining, Pearson *r*^2^ = –0.77, *P* < 0.001. **(L)** Positive correlation between FBXO2 and Ki67 staining, Pearson *r*^2^ = 0.857, *P* < 0.0001.

We showed that knockdown of FBXO2 caused a significant down-regulation of CDK4, CCND1, CCND2, and CCNA1 but not of MCM7, SMC1A, CHEK1, or CDC14B (Figure 7A). The western blotting results showed that knockdown of FBXO2 and overexpression of FBN1 decreased the protein expression of CDK4, Cyclin D1, Cyclin D2, and Cyclin A1 in RL95-2-shFBXO2 cells compared with RL95-2-NC cells. Knockdown of both FBXO2 and FBN1 significantly increased the expression of the aforementioned cell cycle proteins in RL95-2-shFBXO2/shFBN1 cells compared with RL95-2-shFBXO2 cells (Figure 7B). Furthermore, a significant upregulation of ATG4A and ATG4D (proteins involved in autophagic cell death) was observed in the shFBXO2 group compared with the RL95-2-NC group (Figure 7C). We also found that inhibition of cyclin-dependent kinases by palbociclib (PD0332991, which specifically inhibits cyclin-dependent kinases 4 and 6 [CDK4/6]) (Liu et al., 2018) reversed the effects of FBXO2 on proliferation of Ishikawa cells (Figure 7D). We also showed that the inhibition of 3-methyladenine (3-MA, an autophagy specific inhibitor) (Luan et al., 2019) reversed the effects of FBXO2 on proliferation of RL95-2 cells (Figure 7E). Hence, from our results, we proposed that FBXO2/FBN1 was important in promoting EC proliferation via the cell cycle and the autophagy signaling pathway.

### FBXO2 Silencing Inhibits EC Carcinogenicity Through FBN1 *in vivo*

To support the *in vitro* findings, we evaluated the function of FBXO2 in EC using a nude mouse model. Mice were injected subcutaneously with RL95-2-NC, RL95-2-shFBXO2, RL95-2-shFBN1, or RL95-2shFBXO2/shFBN1 stable cell lines at a concentration of 5 × 10^6^ cells in 0.2 ml of medium. Compared with the mice injected with RL95-2-NC cells, those injected with RL95-2-shFBXO2 cells displayed an attenuated rate of tumor growth while knockdown of both FBXO2 and FBN1 rescued this phenotype (Figures 7F,H,I). In addition, weight of tumors derived from the RL95-2-shFBXO2 group was significantly reduced relative to that derived from the RL95-2-NC group. However, tumor weights were much higher with double-knockdown of FBXO2 and FBN1 compared with the RL95-2-shFBXO2 group (Figure 7I). When we used IHC to examine FBXO2, FBN1, and Ki67 expression in tumors, we confirmed that FBXO2 expression was diminished in the RL95-2-shFBXO2 group (Figure 7J, column 2) and that FBN1 was reduced in the RL95-2-shFBN1 group (Figure 7J, column 3). Knockdown of FBXO2 expression resulted in increased expression of FBN1 and decreased expression of Ki67 (Figure 7J, column 2), and knockdown of FBN1 or double-knockdown of FBXO2 and FBN1 resulted in decreased ki67 expression (Figure 7J, column 3 and 4). This indicated that the absence of FBN1 blocked the proliferative role of FBXO2. FBXO2 was inversely correlated with FBN1 expression and positively correlated with Ki67 expression (Figures 7J–L). Collectively, these data indicate that FBXO2 functions as a tumor oncogene, and that it is essential for EC cell growth.

## Discussion

Using multiple complementary approaches, we identified that FBXO2, precisely the C-terminal FBA domain, directly binds to FBN1, leading to its degradation by polyubiquitination, promoting proliferation of EC by inactivating the cell cycle and inhibiting the autophagy signaling pathways.

F-box proteins regulate substrates in diverse biologic processed that control essential aspects of cellular life, including cell growth, development and differentiation, and cell survival and death. And dysregulation of F-box protein-mediated ubiquitylation has been demonstrated in various types of cancers (Frescas and Pagano, 2008; Crusio et al., 2010; Inuzuka et al., 2011). FBXO2, a member of the human F-box family, is a cytoplasmic protein and a neuron-enriched ubiquitin ligase substrate adaptor protein (Nelson et al., 2006) that binds the signature *N*-linked high-mannose glycan moiety of glycoproteins, and mediates the ubiquitination of ER glycoproteins in the ER-associated degradation system (Schroder and Kaufman, 2005). Furthermore, the FBXO2 variant rs99614 C allele was found to decrease the risk for Parkinson’s disease in mainland Han Chinese (Yuan et al., 2017). FBXO2, along with the presence of an F box domain was also highly enriched in the nervous system, and involved in cell cycle regulation by directly interacting with Skp1p (Erhardt et al., 1998). In addition, the FBA domain of FBXO2 was equally essential for its activity of glycoprotein-recognizing activity. Liu et al. (2017) proved that FBXO2, along with the Fox-associated domain (FBA) activity, interacted with the insulin receptor to enhance its ubiquitination-mediated protein degradation. FBXO2 also promoted the degradation of Epstein-Barr virus glycoprotein though its sugar-binding domain, and decreased the entry of the virus (Zhang et al., 2018). Investigators have in recent years studies the role of FBXO2 in various cancers. For example, FBXO2 was reported to regulate the EMT signaling pathway in gastric cancer (Sun et al., 2018) and high expression levels if FBXO2 correlated with colorectal cancer metastasis, such that it is now used as a reliable predictor of poor prognosis in colorectal cancer patients (Wei et al., 2018).

In this study, we found that FBXO2 was significantly up-regulated in EC compared with normal endometrium based on RNA-seq. We also determined the clinical relevance of FBXO2 in EC and demonstrated that FBXO2 levels were positively associated with tumor stage, grade, histologic type, and poor survival. Furthermore, FBXO2 showed high expression in EC samples compared with para-carcinoma tissues, and was positively associated with muscular infiltration and Ki67 staining (Figure 1). We also demonstrated that knockdown of FBXO2 inhibited EC cells proliferation, and that overexpression of FBXO2 promoted EC cells proliferation (Figure 2). We used liquid chromatograph-tandem mass spectrometry and found that FBN1 was one of the substrates regulated by FBXO2 ubiquitination. We also demonstrated that FBN1 directly interacted with FBXO2 and showed ubiquitination-mediated degradation by FBXO2 (Figures 3, 4). FBN1 is reported to be located on chromosome 15 and encodes a type of large extracellular matrix glycoprotein named fibrillin 1 (Biery et al., 1999). FBN1 can form threadlike filaments, and microfibrils, providing structural support for tissues, and forms elastic fibers in skin and blood vessels (Isogai et al., 2003; Massam-Wu et al., 2010). Prior studies have revealed that the FBN1 gene promoter was hypermethylated in colorectal cancer and endothelial tumor cells (Hellebrekers et al., 2007; Lind et al., 2011). Lack of the FBN1 protein, then, may play a potential role in tumors and the FBN1 gene may be considered an essential tumor-suppressor gene. In addition, plasma FBN1 has proven to be one promising biomarker in aiding the diagnosis of spontaneous coronary artery dissection (Zhu et al., 2018; Hui et al., 2020). Our next approach showed that FBN1 was required for FBXO2 to exert functional impacts on EC proliferation (Figure 5). The physiologic role of FBXO2 was further revealed with nude mice experiments (Figure 7), which suggested that inhibiting the expression or activity of FBXO2 represents a potential therapeutic role for the precise treatment of EC.

Our data also indicated that aberrant expression of FBXO2 was attributed to the cell cycle and autophagy signaling pathway (Figures 6, 7). The cell cycle is the sequence of events by which a cell duplicates its genome, and grows, and divides. Cyclin-dependent kinases and other kinases comprise the key cell cycle transitions. Dysregulation of the cell cycle is a hallmark of cancer that leads to aberrant cellular proliferation (Poon, 2016; Bonelli et al., 2019). In our study, knockdown of FBXO2 or overexpression of FBN1 led to significant down-regulation of CDK1, CyclinD1, CyclinD2, and CyclinA1. Furthermore, knockdown of both FBXO2 and FBN1 reversed levels of the aforementioned proteins. Importantly, the CDK4/6 inhibitor, Palbociclib, reversed the effects of FBXO2 on proliferation in Ishikawa cells (Figure 7). Pharmacologic inhibitors of CDK4/6 have recently shown the promising activity in patients with cancers. Based on our data, we suggest that those patients showing a high expression of FBXO2 are potential candidates for CDK4/6 inhibitor treatment. Autophagy, a conserved self-catabolic process, enables the cells to remove and recycle the cytoplasmic contents, such as toxic molecules and invading microorganisms, and is comprised of five stages: initiation, nucleation, maturation, fusion with the lysosome, and degradation (Shintani and Klionsky, 2004; Saha et al., 2018). This process is strictly coordinated by autophagic core proteins encoded by more than 30 AuTophaGy-related (ATG) genes. ATG4 (consisting of four homologs: ATG4A, ATG4B, ATG4C, and ATG4D) plays an essential role in the regulation of the Atg8/microtubule-associated protein 1A/1B-light chain 3 (LC3) lipid conjugation system (Fu et al., 2019). In the present study, knockdown of FBXO2 up-regulated ATG4A and ATG4D, suggesting that silencing FBXO2 may activate ATG cell death. In addition, 3-MA (one autophagy specific inhibitor) reversed the effects of FBXO2 on the proliferation of RL95-2 cells (Figure 7).

In all, our results have identified FBXO2 as a functional ubiquitin enzyme for FBN1 and provided valuable insights into a potential role of FBXO2 in EC treatment. FBXO2 may be serve as a potential therapeutic target for EC precise treatment.

## Data Availability Statement

The datasets presented in this study can be found in online repositories. The names of the repository/repositories and accession number(s) can be found in the article/Supplementary Material.

## Ethics Statement

The studies involving human participants were reviewed and approved by this present study was approved by the Ethics Committee of Ruijin Hospital affiliated to Shanghai Jiao Tong University School of Medicine and Obstetrics and Gynecology Hospital of Fudan University. The patients/participants provided their written informed consent to participate in this study. The animal study was reviewed and approved by all animals were handled according to the Guide for the Care and Use of Laboratory Animals’ and the Principles for the Utilization and Care of Vertebrate Animals.

## Author Contributions

WF: design of this study. XC, FJ, YW, and JZ: acquisition of data. XC, FJ, QC, and XW: data analysis. XC, FJ, and JS: drafting the article. WF and NJ: grammar and language modification. All authors contributed to the article and approved the submitted version.

## Conflict of Interest

The authors declare that the research was conducted in the absence of any commercial or financial relationships that could be construed as a potential conflict of interest.

## Footnotes

1 www.cellbank.org.cn
