# Supplementary material for: FBXO2 Promotes Proliferation of Endometrial Cancer by Ubiquitin-Mediated Degradation of FBN1 in the Regulation of the Cell Cycle and the Autophagy Pathway
Source: Front Cell Dev Biol. 2020 Aug 31;8:843. doi: 10.3389/fcell.2020.00843 (PMC7487413; doi:10.3389/fcell.2020.00843)
Supplement: Supplementary file 4 [file Data_Sheet_1.zip › STR reports/HEC-1A STR.pdf]

细胞遗传质量鉴定检测

Cell Line Authentication Service

STR 基因型检测报告

送检单位：复旦大学附属妇产科医院

检品名称：细胞系

委托单位：上海翼和应用生物技术有限公司

报告日期：2019-01-02

上海翼和应

报告说明

1. 本报告只对送检的来样负责。
2. 检验报告上的检验结果和检验单位名称，未经同意不得用于广告、评优及商业宣传。
3. 对本报告有异议，请于收到报告之日起十五日内以书面方式提出，逾期不予受理。
4. 对纸质检验报告涂改、增删，或未加盖检验单位印章的复印件均无效。

样品信息

样品编号：

客户样本编号	公司编号
HEC-1A	20181224-01

样品数量： 1

样品性状： 细胞系

检测项目： STR

送检单位： **复旦大学附属妇产科医院**

检测方法： 用 Axygen 的基因组抽提试剂盒提取 DNA，采用 21- STR 扩增方案扩增，在 ABI 3730XL 型遗传分析仪上对 STR 位点和性别基因 Amelogenin 进行检测。

检测结果

(一) 检验基本情况

公司编号	多等位基因	匹配细胞系	细胞库	EV 值	匹配说明
20181224-01	无	HEC-1-A	EXPASY	0.92	基本匹配

样本基因型检验结果

- 多等位基因指三等位及以上基因现象。
- 本次检测各细胞分型结果良好。

(二) 各样本描述

- 20181224-01：该株细胞 DNA 分型在细胞系检索中找到**基本匹配**的细胞系，EXPASY 数据库显示细胞名为 **HEC-1-A**，细胞号对应 **CVCL_0293**。本次检测在该细胞系中**没有发现多等位基因**。

  备注：待测细胞系与收录于 ATCC, DSMZ, JCRB 和 RIKEN 数据库的细胞系 STR 数据进行比对，未收录于以上细胞库的细胞系将无法匹配。

（三）样本分型结果

细胞的 STR 位点和 Amelogenin 位点的基因分型结果						
Loci	送检细胞 STR 信息			细胞库细胞 STR 信息		
	送检细胞名：HEC-1A			细胞库细胞名：HEC-1-A		
	Allele1	Allele2	Allele3	Allele1	Allele2	Allele3
D5S818	11	15		11	15	
D13S317	11	11		11	11	
D7S820	9	11		9	11	
D16S539	12	12		12	12	
VWA	18	19		18	19	
TH01	6	6		6	7	
AMEL	X	X		X	X	
TPOX	8	8		8	11	
CSF1PO	10	12		10	12	
D12S391	19	20				
FGA	21	22				
D2S1338	19	19				
D21S11	30	31				
D18S51	16	21				
D8S1179	13	14				
D3S1358	15	15				
D6S1043	12	18				
PENTAE	11	11				
D19S433	13.1	13.1				
PENTAD	9	13				
D1S1656	18.3	18.3				

其他说明

(一) 分型方案及位点分布

	方案 1	方案 2	方案 3	方案 4
1	D3S1358	D8S1179	D19S433	AMEL
2	VWA	D21S11	TH01	D1S1656
3	D7S820	D16S539	D13S317	D5S818
4	CSF1PO	D2S1338	TPOX	D12S391
5	PENTAE	PENTAD	D18S51	FGA
6			D6S1043	

实验方案及位点

(二) STR 数据库比对

本公司采用 DSMZ tools 进行细胞系比对，其中包含来自于 ATCC, DSMZ, JCRB 和 RIKEN 数据库的 2455 个细胞系 STR 数据。如果待检测细胞未收录于以上细胞库或这是自行建立的新细胞系将无法进行比对，用户需根据细胞分型结果自行与其他数据库进行比对。

(三) 文献引用参考

1．Authentication testing of HEK 293T and HeLa cell lines have been performed by Shanghai Biowing Applied Biotechnology Co.,Ltd via STR profiling. STR profiles match the standards rec ommended for HEK 293T and HeLa cell lines authentication

2．AGS, NCI-N87, HGC-27 and HEK293 were STR-authenticated on Dec. 8, 2015 by Shanghai Biowing Applied Biotechnology Co. LTD, Shanghai, China

主要实验人员：张佳男
复核人: 钱宁
负责人：白杨

上海翼和应用生物技术有限公司

签发日期：2019-01-02
